# A Review on the Evolving Roles of MiRNA-Based Technologies in Diagnosing and Treating Heart Failure

**DOI:** 10.3390/cells10113191

**Published:** 2021-11-16

**Authors:** Peter J. Kennel, P. Christian Schulze

**Affiliations:** Department of Medicine I, Division of Cardiology, University Hospital Jena, Friedrich-Schiller-University Jena, Am Klinikum 1, 07747 Jena, Germany; peter.kennel@uni-jena.de

**Keywords:** MicroRNA, miRNA, cardiac biomarker, heart failure, antagomir

## Abstract

MiRNA-regulated processes are pivotal in cardiovascular homeostasis and disease. These short non-coding RNAs have ideal properties that could be utilized as potential biomarkers; moreover, their functions as post-transcriptional regulators of mRNA make them interesting therapeutic targets. In this review, we summarize the current state of miRNA-based biomarkers in a variety of diseases leading to heart failure, as well as provide an outlook on developing miRNA-based therapies in the heart failure field.

## 1. Introduction

MiRNAs are a class of short non-coding RNAs that are essential to virtually every cellular process, by acting as post-transcriptional regulators of mRNA. Our understanding of miRNA functioning is rapidly evolving, and its mechanisms are highly complex, with miRNA target and effect interactions depending on multiple factors, such as the cellular compartmental location of miRNAs, the ratio of miRNAs and target mRNA abundance, and binding affinity of miRNAs and binding sites. Most commonly, miRNAs interact with the 3′-UTR, also termed microRNA response element (MRE) of mRNA transcripts, and lead to target mRNA degradation and translational repression [1]. The majority of identified miRNAs are transcribed from intron or intergenic regions, and are regulated by their own respective promotors. Maturing miRNAs undergo several processing steps, resulting in single-strand ribonucleotides of usually around 22 bp length. The nomenclature distinguishes between 5p strands, arising from the 5′ end of the pre-miRNA duplex and the 3p strand from the 3′ end [1]. Mature miRNAs exhibit regulatory functions on mRNA translation via RNA-induced silencing complexes (RISC), which are comprised of the respective miRNA strands and proteins of the argonaute protein family, among other components. The RISC then uses this miRNA guide strand to target the 3’-UTR of mRNA transcripts [2]. MiRNA-mediated gene silencing is mediated by recruitment of RNA-deadenylation enzyme complexes by the RISC, exposing the mRNA to rapid degradation.

MiRNAs, acting as key regulators for cell development, differentiation, and homeostasis, could act as autocrine, paracrine, or remote signaling mechanisms, as miRNAs can be secreted into the systemic circulation, and transported (protein-bound or via vesicles). This has led to efforts in developing miRNA-based biomarkers for a great variety of diseases, as well as miRNA-based research techniques to modulate gene expression, with the aim to develop miRNA-based therapies.

In the cardiovascular field, knowledge on the role of miRNAs in cardiac homeostasis and disease has rapidly evolved, and miRNA-regulated pathways have been identified for most myocardial processes. Examples of two “classic” cardiac miRNAs whose roles in cardiovascular disease have been well characterized are miR-1 and miR-133. These miRNAs are most abundant in myocardial tissue, and have been implicated in cardiogenesis, and in the adult heart—in myocardial remodeling, hypertrophy, and conduction system disturbances [3,4,5].

The field of miRNA research is rapidly evolving. To date, over 2000 miRNAs have been described in humans; however, relatively few have been put into a functional context [6,7].

In this review, we outline the current understanding of the role of miRNAs in the pathology of cardiomyopathies and the potential of miRNA-based diagnostic tools. Then, we summarize the current state of development of miRNA-based cardiovascular therapeutics as well as future challenges in cardiovascular miRNA research.

## 2. MiRNA-Based Diagnostics

Many miRNAs are secreted into the systemic circulation via exosomes and microvesicles, or are lipid-bound. Harnessing the favorable characteristics of circulating miRNA complexes, which are quite stable and easily extracted and amplified, research efforts have led to the characterization of miRNA profiles in different types of cardiomyopathies. Various miRNAs have been described as potential biomarkers for ischemic heart disease, as well as for various non-ischemic cardiomyopathies, such as HCM, infiltrative heart disease, or inflammatory cardiomyopathies (CMP), as outlined below and in Table 1.

### 2.1. Myocardial Ischemia

MiRNAs have been explored as potential biomarkers, indicating acute coronary syndrome. For these studies, myocardium-specific circulating miRNAs have been measured. Using bioinformatic techniques, such as functional enrichment analysis, attempts to identify candidate miRNAs for acute coronary syndrome (ACS) biomarkers have been made [22], and using these methods, among others, a confusingly large number of miRNAs were proposed to be involved in myocardial injury. The large body of studies (of varying quality/technical approaches) may be distilled into a few miRNAs, which were reproducible in several studies, providing some mechanistic insights. These are miR-133, miR-499; miR-1, and miR-208. All of these are highly expressed in myocardium, albeit not cardio-specific.

#### 2.1.1. MiR-499

MiR-499 is one of the most myocardium-specific miRNAs currently known. Several studies have assessed this noncoding RNA for its utility as a biomarker for ACS. In a study comparing sensitivity and specificity or miR-499 elevation in a cohort of ACS patients compared to non-ACS chest pain patients, miR-499 showed similar power (0.92) as hs-Troponin (0.925) for the prediction of myocardial infarction (MI) [8]. A meta-analysis that included 8 studies and a total of 1634 patients reported an AUC of 0.958 for miR-499 as a biomarker of myocardial infarction [23]. Of note, the study design varied widely among studies and the authors emphasized that this apparent systematic difference, or publication bias, limited the validity of the findings. Another (more recent) meta-analysis that included 3816 participants reported a similar excellent performance of miR-499 to indicate ACS, but with similar concerns about the heterogeneity of the study designs, mainly introduced by the inclusion of healthy subjects as controls [24]. Consequently, in a sub-group analysis that excluded studies with healthy controls, sensitivity of miR-499 was much lower compared to the initial analysis (sensitivity 0.57, 95% CI: 0.21–0.93), specificity (0.91, 95% CI: 0.80–1.00), versus sensitivity 0.91, 95% CI: 0.83–1.00; specificity (0.99, 95% CI: 0.96–1.00), respectively.

#### 2.1.2. MiR-133

MiRNA-133 is highly expressed in muscle tissue and is not cardiac-specific. Several studies have assessed the sensitivity and specificity of this miRNA in ACS, with some reporting excellent validity. Kubawara et al. 2011: non-ACS (*n* = 42) vs. ACS (*n* = 29): AUC 0.932 [25]; Peng et al. 2014: non-ACS (*n* = 110) vs. ACS (*n* = 76): AUC 0.912 [26]. A recent meta-analysis that included eight studies reported a pooled AUC of 0.73 (95% CI 0.68–0.79). Of note, five out of eight studies yielded AUC values greater than 0.86; however, given the relatively small sample size, the overall weight was reduced [27]. Of note, other well-conducted studies have reported far lower sensitivities and specificities for this miRNA, placing the utility of miR-133 in question [28]. One possible explanation for these varying results may lie in the heterogeneous study design, such as the choice of control groups. When miR-133 was tested, in comparison to healthy controls, it showed a significantly higher performance when compared to a control group of patients with chest pain [27]. Therefore, while being one of the most-studied miRNA-based biomarkers for ACS, its utility remains to be better characterized.

#### 2.1.3. MiR-1

MiR-1 is a microRNA that is highly expressed in muscle tissue; it has been implicated in many basic myocardial functions, such as differentiation of cardiac precursor cells and the regulation of calcium cycling [29]. It has been shown that miR-1 (among other cardiac miRs) is released into the circulation via exosomes and activates myeloid progenitor cells, mounting a systemic response to myocardial injury [30]. MiR-1 has been frequently studied for its potential as an ACS-biomarker and several meta-analyses are now available characterizing its diagnostic accuracy. In a meta-analysis that included 11 studies, Zhai et al. reported a sensitivity of 0.78 (95% CI: 0.71–0.84), specificity: 0.86 (95% CI: 0.77–0.91), AUC: 0.88 (95% CI: 0.85–0.90) for miR-1. As noted previously, there are similar concerns about a potential bias caused by the heterogeneity of the available studies (quality of the included studies, age, gender proportion, and sampling criteria) [31]. In a recent study with well-defined inclusion criteria, including 337 patients presenting with chest pain, Su et al. reported a similar performance between miR-1 and troponin-I when sampled within 3 h of CP onset (with no high-sensitivity troponin test), and that higher miR-1 levels were associated with a worse prognosis [32].

#### 2.1.4. MiR-208

MiR-208 is a miRNA that is highly expressed in myocardium [33]. A recent meta-analysis that included 13 studies evaluated the diagnostic value of miRNA-208, and reported the performance of pooled miR-208 (a/b) sensitivity and specificity at 0.83 (95% CI: 0.74–0.89) and 0.98 (95% CI: 0.88–0.99), respectively, and SROC 0.94 (95% CI: 0.91–0.95), albeit with a significant potential for publication bias [31]. There appears to be a significant association between elevated miR-208 levels and higher mortality in AMI, compared to other myomiRs [34,35].

A recently published (well-designed) study by Schulte et al., comparing performance parameters between miRNA biomarker candidates and (hs-)troponin, concluded that, in an acute MI cohort, cardiac miRNAs (miR-499 and miR-208b) showed comparable AUC values to hs-troponin, but with an overall lower sensitivity than the gold standard. In a multi-biomarker approach, including muscle-enriched miRNAs (miR-1 and miR-133a), hs-troponin, and another protein marker, cMyBP-C, returned the highest AUC for MI. (AUC 96.9 vs. AUC 92.5 for hs-troponin) [8].

MiRNA-panels have also been tested for their prognostic value in assessing cardiovascular mortality. In a recent study by Keller et al. [36], which included 178 patients from a study cohort representing a western European primary care patient population, as well as a validation cohort comprised of 129 individuals without known cardiovascular disease, of which 64 individuals died within a year after enrolment, and were matched to a control group of surviving patients, an miRNA-panel consisting of 5 miRNAs (MiR-34a, miR-223, miR-378, miR-499, and miR-133) was significantly associated with cardiovascular mortality ((HR 3.0 (95% (CI): 1.09–8.43; *p* = 0.034; validation cohort: HR of 1.31 (95% CI: 1.03–1.66; *p* = 0.03). The authors concluded that conventional risk models, such as the Framingham or the SCORE risk model, were significantly improved by including this miRNA panel to predict mortality. Various other studies have proposed other miRNA signatures with promising prognostic value characteristics [37,38,39]; however, to date, none of these have been established in clinical practice.

In conclusion, the clinical usefulness of miRNA-based biomarkers for ACS remains limited for the following reasons. Currently, established miRNAs with biomarker-potential appear to have a lower sensitivity than the current gold standard of (hs)-Troponin. Technologies that would perform—with a point-of care test or with rapid turnaround, as mandated by the clinical demand for a test to rule in/out ACS—are currently not established. A multi-biomarker assay, including certain miRNAs, may provide a higher performance than traditional biomarkers [31]; however, cost and complexity of such an assay would have to be weighed against the clinical significance of a marginally improved diagnostic test. One interesting aspect of measuring circulating miRNA levels involves kinetics, as it was shown that cardiac miRNAs remain in circulation in the 24 h after myocardial injury occurs [8].

### 2.2. Non-Ischemic Cardiomyopathies—NICM

A biomarker that could aid in the differentiation of non-ischemic cardiomyopathies would be of great value for the workup of newly diagnosed heart failure. Several studies were conducted to identify potential miRNAs that would indicate a specific etiology of myocardial failure. While low-level elevated troponin is a common and non-specific find for most cardiomyopathies, current research efforts are looking to identify CMP-pathology type specific miRNAs. Several murine knockout models have proven the functional role of miRNAs in the pathogenesis of HF, with miRNA knockout models available for phenotypic HCM [40], dilated cardiomyopathy (DCM) [41], and many more miRNA knockout murine models established, presenting phenotypically with various myocardial and vascular deficits [42]. With the advent of CRISPR technology, cardiac-specific genomic editing has been made possible with the potential to modify whole miRNA clusters, to establish new HF models and explore the role of miRNAs in the pathogenesis of HF more easily [43,44].

#### 2.2.1. Hypertrophic Cardiomyopathy—HCM

HCM is a hereditary, autosomal dominant cardiomyopathy with a heterogeneous phenotype and variable penetrance. Several genetic mutations of the sarcomere have been identified, and their numbers have increased rapidly with novel sequencing technologies [45]. Consequently, miRNA profiling has also been performed in HCM patients, which has led to the identification of potential roles for miRNA-based biomarkers for HCM. The presence and degree of myocardial fibrosis, which is usually diagnosed via cardiac MRI, is clinically relevant for the prognosis and management of HCM patients. To assess the role of miRNAs as markers for fibrosis in HCM patients, Lu et al. used a miRNA microarray in serum samples of 55 HCM patients and identified a cluster of differentially expressed miRNAs in HCM patients with fibrosis compared to those without endomyocardial fibrosis. While individual miRNAs had moderate diagnostic values for diffuse myocardial fibrosis (AUC: 0.663–0.742), the diagnostic value was greatly improved (AUC: 0.87) for a combination of eight miRNAs [46]. In a smaller study that included a patient cohort with established sarcomere mutations diagnostic for HCM (23 patients with hypertrophic non-obstructive CMP (HNCM), 28 patients with hypertrophic obstructive cardiomyopathy (HOCM), Derda et al. identified miR-29a as microRNA of particular interest in HCM. Levels of this miRNA were increased in patients with HOCM and correlated with severity of cardiac hypertrophy, in contrast to HNCM. Interestingly, in this small study, the authors were able to show that hypertrophy caused by aortic stenosis could be distinguished from HCM by miR-29a/c, with fairly good performance (AUC 0.76; *p* < 0.002). [9] Recently, Wang et al. reported on a systematic study of miRNA profiling in HCM, utilizing miRNA profiles from the Gene Expression Omnibus (GEO) database to identify differentially expressed miRNAs between HCM and normal controls [10]. Using this unbiased approach, the authors identified 10 upregulated and 14 downregulated differentially expressed miRNAs, and subsequently identified a miRNA cluster of 4 miRNAs (miR-373, miR-371-3p, miR-34b, and miR-452), which showed excellent performance in separating HCM from controls in a logistic regression model [10].

#### 2.2.2. Cardiac Amyloidosis

Extracellular deposition of amyloid protein in the heart is mainly caused by monoclonal immunoglobulin light chains (AL) or transthyretin (ATTR) fibril deposition. While non-invasive tools often suffice to establish a diagnosis of cardiac amyloidosis, tissue biopsy is sometimes still required, in particular, for suspected AL amyloid. miRNA-based approaches for the workup of cardiac amyloidosis could therefore be a valuable diagnostic tool. To date, only a few smaller studies are available. In a study that included blood samples from 13 hereditary TTR mutation patients (ATTRm), 11 senile cardiac amyloidosis patients (wild type TTR; ATTRwt), as well as healthy controls and heart failure patients, Derda et al. identified a significant upregulation of miR-339-3p in ATTRwt patients compared to the other cohorts [11]. To date, the function of this miRNA remains poorly characterized and it is not tissue-specific. Serum miRNA expression profiling has been performed in patients with ATTRm: comparing miRNA expression profiles between hereditary ATTR patients (presenting with typical symptoms, such as neuropathy and CMP) with asymptomatic ATTRm carriers, the authors identified miR-150-5p as a potential biomarker to differentiate ATTRm patients from asymptomatic TTR carriers (AUC: 0.9728; *p* < 0.0001) [12]. Of note, recently, as a major advancement in the field of RNA-based technologies, TTR is one of the first diseases for which RNA-based therapeutics have been approved (polyneuropathy in ATTRm) [47,48].

#### 2.2.3. Arrhythmogenic Right Ventricular Cardiomyopathy—ARVC

ARVC is characterized by often life-threatening arrhythmias and myocardial structural abnormalities, such as fibrofatty replacement of myocardium, in, most often, the right ventricle (although left-sided involvement is common). ARVC is a rare disease and requires a high level of clinical suspicion. Genetic mutations in desmosomal proteins have been recognized as causative for ARVC (plakophilin, plakoglobin, desmoplakin, desmoglein, and desmocollin protein). The diagnostic workup includes cardiac MRI and genetic testing. miRNA profiling in this complex disease can offer valuable insights into its pathogenesis and possibly improve early detection. In a study involving pooled myocardial RNA samples of 24 ARVC patients, global microRNAs expression profiling (measuring tissue levels of 1078 microRNAs in the assay) yielded miRNA signature of 21 miRNAs in ARVC compared to healthy control samples, and suggested a link between this rare cardiomyopathy and the Wnt- and Hippo pathways [15]. Other recent studies have reported potential ARVC-specific miRNA signatures (miR-144-3p, miR-145-5p, miR-185-5p, miR-494 [16,17]. A murine model for arrhythmogenic cardiomyopathy is available and has offered mechanistic insights into the role of miRNAs in the structural changes in this CMP, implicating miR-708-5p, miR-217-5p, and miR-499-5p, and their regulatory functioning on the Wnt-pathway in the pathology of arrhythmogenic cardiomyopathy [49]. Marinas et al., in the largest cohort to date, and in an unbiased approach that included samples from over 90 ARVC patients, reported a miRNA signature of miR-122-5p, miR-133a-3p, miR-133b, miR-142-3p, miR-182-5p, and miR-183-5p, with high discriminatory diagnostic power to distinguish between ARVC patients and controls. (AUC: 0.995) [18]. As with many studies in the miRNA-field, sample sizes are still generally small, and study designs and methods are heterogeneous; to date, the results rarely show an overlap in single miRNA or miRNA signatures between studies. Nevertheless, currently available studies were able to elucidate important aspects of the pathology of arrhythmogenic cardiomyopathies.

#### 2.2.4. Inflammatory Cardiomyopathies

This is a heterogeneous group of inflammatory cardiac processes, but a common cause of resulting dilated CMP. Causes of myocarditis include infections as well as autoimmune processes. While, oftentimes, the exact etiology of the inflammatory process cannot definitively be determined, several well-defined diseases can be delimited, such as giant-cell myocarditis, cardiac sarcoidosis, or specific viral myocarditis [50].

*Viral myocarditis*: the knowledge on the role of miRNAs in regulating inflammatory cascades is rapidly evolving. miRNA profiling of myocardial biopsy samples for some specific viral infections, such as Coxsackie virus myocarditis, have been reported, and show complex differentially expressed miRNA profiles. Since miRNAs have regulatory functions on complex disease processes, these differentially expressed profiles possibly indicate general inflammatory cascades, apoptosis and fibrosis, rather than miRNA profile specific to a pathogen. Nevertheless, a specific miRNA to indicate acute myocarditis would be of great value, as, to date, the diagnosis is difficult to attain and at times still requires invasive endomyocardial biopsy. Most recently, in an intriguing approach, Blanco-Domínguez identified a type 17 helper T (Th17) cell-synthesized miRNA, miR-Chr8:96, which showed promising sensitivity and specificity for distinguishing patients with acute myocarditis from those with myocardial infarction (ROC: 0.927 (95% CI: 0.879–0.975) [19]. Therefore, lymphocyte-derived miRNA-profiling could be a promising way forward towards distinguishing inflammatory CMPs.

*Cardiac sarcoidosis:* sarcoidosis is a systemic disease, with still incompletely understood etiology and a multifaceted clinical presentation. Typical presenting symptoms, such as often life-threatening arrhythmias, heart failure, or myocardial scarring without coronary disease, or accompanying non-cardiac symptoms, often initiate the workup for this complex disease, including cardiac imaging and endomyocardial biopsy. Few studies have reported potential roles of miRNAs in cardiac sarcoidosis. Crouser et al. isolated exosomal miRNA from 21 patients with cardiac sarcoidosis and compared their miRNA profile to patients with sarcoidosis without cardiac involvement (*n* = 21) and healthy controls. Differentially expressed miRNAs identified via a next-generation sequencing approach were validated with PCR and resulted in three miRNAs (miR-7-1-3p, miR-32-3p, and miR-211-5p) that were significantly differently expressed compared to controls, and between CS and sarcoidosis, without cardiac involvement. Moreover, miR-32-3p and miR-211-5p) were found to have higher concentrations in the non-CS group compared with the CS group, and miR-7-1-3p was significantly elevated in patients with CS [13]. Another small study by Fujiwara et al. was performed, consisting of an exploratory group of serum samples from 5 CS patients, with which genome-wide expression profiling was conducted, yielding 12 differentially expressed candidate miRNAs compared to healthy controls. These miRNAs were then tested via PCR in a confirmatory set of 15 sarcoidosis patients and yielded two miRNAs—miR-126 and miR-223—significantly higher in sarcoidosis patients compared to healthy individuals [14]. These miRNAs have been implicated in various immunological processes [51,52]. In a similar experiment to the previously described studies, most recently, Crouser et al. performed next-generation miRNA-sequencing on exosome-derived samples of patients with CS, AMI, and healthy controls (*n* = 10 in each group), reporting differentially expressed miRNA profiles, distinguishing the cardiac pathologies from each other and control [53].

*Giant cell myocarditis:* this is a rare and often fulminant, rapidly progressive type of autoimmune mediated myocarditis, typically presenting with rapidly progressing heart failure, leading to cardiogenic shock and life-threatening arrhythmias. The pathology of giant-cell myocarditis is still incompletely understood; to date, no studies on miRNA profiles are currently available.

#### 2.2.5. Stress CMP/Takotsubo CMP

Stress CMP is the diagnosis of exclusion in patients with regional ventricular dysfunction, in the absence of significant CAD. Catecholamine surge and microvascular/endothelial dysfunction are suspected to play a role in the pathology. Clinically, stress CMP is often difficult to diagnose and coronary angiography is usually necessary. Therefore, novel biomarkers that could reliably distinguish stress CMP would be of great clinical benefit. In a study that included patients diagnosed with stress CMP (*n* = 36), STEMI (*n* = 27), and healthy controls (*n* = 28), Jaguszewski et al. performed miRNA profiling and subsequent confirmatory PCR of candidate miRNAs. For the stress CMP group, a miRNA cluster comprised of miR-16, miR-26a, miR-1, and miR-133a differentiated stress CMP from healthy subjects with promising performance parameters (AUC: 0.835, 95% CI 0.733–0.937, *p* < 0.0001) and from STEMI patients (AUC: 0.881, 95% CI 0.793–0.968, *p* < 0.0001) [20]. Of note, mechanistic data on these two miRNAs from an animal model of stress CMP have recently been reported, demonstrating miR-16 and miR-26a mediated regional myocardial hypokinesis in concert with adrenaline exposure, with a link to reductions of the calcium channel subunit and g-protein receptor protein expression [21]. Therefore, miR-16 and miR-26 could be important targets for the development of miRNA-based biomarkers and possibly even therapeutic approaches for stress CMPs.

## 3. Outlook: Developing miRNA-Based Therapies in the Heart Failure Field

miRNAs regulate gene expression at the mRNA level. Deregulation of miRNAs has been linked to a great variety of diseases and, therefore, the development of miRNA-based therapies has become the focus of research efforts in the field. Delivery of selected miRNAs to specific tissues to target multiple mRNAs that are altered in the respective disease or silencing pathologically overexpressed miRNAs via “antagomiRs” or “antimiRs”, are potential approaches [54]. Currently, there are few miRNA-based therapeutics in early clinical trials, for a variety of diseases, such as lademirsen (miR-21, Alport syndrome); remlarsen (miR-29, keloid), cobomarsen (antagomir-155, T-cell lymphoma), or miravirsen (miR-122 antagomir, hepatitis C). Regarding the development of miRNA-based therapeutics—few short-interfering RNA-based drugs have already reached clinical approval: siRNAs and miRNAs share many properties, both acting as gene silencers via mRNA interference and degradation. However, miRNAs can bind to multiple mRNAs and, therefore, influence translation of multiple mRNA targets [55]. siRNAs, on the other hand, are highly specific to one target mRNA. To date, three siRNA-based therapies have been approved (patisiran, givosiran, and lumasiran), with many more in development.

The promise (and challenge) of miRNA based-therapies is therefore the complexity of pleiotropic miRNA effects, possibly influencing cascades of mRNAs for translational changes. While our understanding of miRNAs in cardiovascular disease processes is rapidly expanding, the complex interactions and effects of these small RNA molecules in different disease states is, overall, still only marginally understood. An intriguing path forward is our growing knowledge on the implications of miRNA-regulated (more global) pathologic processes, such as cardiac fibrosis [56] hypertrophy/dilation [57,58], or cardiomyocyte cell death signaling [59], regardless of the specific culprit. For example, while a particular “insult” leading to a dilated cardiomyopathy can be manifold, miRNAs appear to be involved in the resulting phenotype of DCM downstream [58]. Similarly, while cardiac fibrosis and scarring may be caused by coronary artery disease, or a disease on the inflammatory CMP spectrum, the resulting myocardial fibrosis may be mediated by similar miRNA signaling. Manipulating these miRNAs may offer the opportunity to halt or possibly even reverse these pathological changes in the failing heart.

Specifically, in the cardiovascular field, only recently was the first phase-I clinical study initiated based on an antimiR-drug: miR-132 levels were increased in patients with heart failure (HF) and mechanistic data hint toward a role, if this miRNA in cardiac fibrosis and remodeling processes. In one of the first miRNA-based therapeutic trials in the cardiovascular disease field, the miR-132 inhibitor CDR132L was administered in a randomized trial that included 28 patients—CDR132L (*n* = 20) or placebo (*n* = 8) [60]. Inclusion criteria were patients 30–80 years old with NYHA class 1–3 chronic HF with LVEF between ≥30% and <50%. Follow-up was 4 months. The study assessed for safety and side effect profiles and reported this drug to be safe and well tolerated, without apparent dose-limiting toxicity. Although it was not designed to report other outcomes—the authors noted a (statistically not significant) NT-proBNP reduction in the treatment group and a significant QRS narrowing, which the authors interpreted as a beneficial effect of the antagomiR on cardiac fibrosis [60]. In another small “first-in human” study, an antimiR to miR-92a-3p was infused in five healthy adults and two placebo controls. miR-92 was associated with endothelial dysfunction and repair, as well as effects in myocardial ischemia models. The antimiR effectively reduced circulating miR-92 levels and led to an increase in miR-92 targets, such as ITGA5, an integrin protein, and various immunological regulatory processes [61]. In the pre-clinical arena, a large number of animal studies on miRNA therapeutics in heart disease are published and/or underway, investigating certain target miRNA-mimics or antagomiRs in a variety of cardiac conditions, such as ischemic heart disease, hypertrophy, or arrhythmias [62]. The design and initiation of further early-stage clinical trials of miRNA-based therapeutics for cardiac conditions are eagerly awaited. In particular, several target miRNAs to modulate fibrosis in non-cardiac conditions show early promising results and may soon be tested for their potential in heart disease. (miR-21, lademirsen); remlarsen (miR-29) [62].

**Figure 1:**
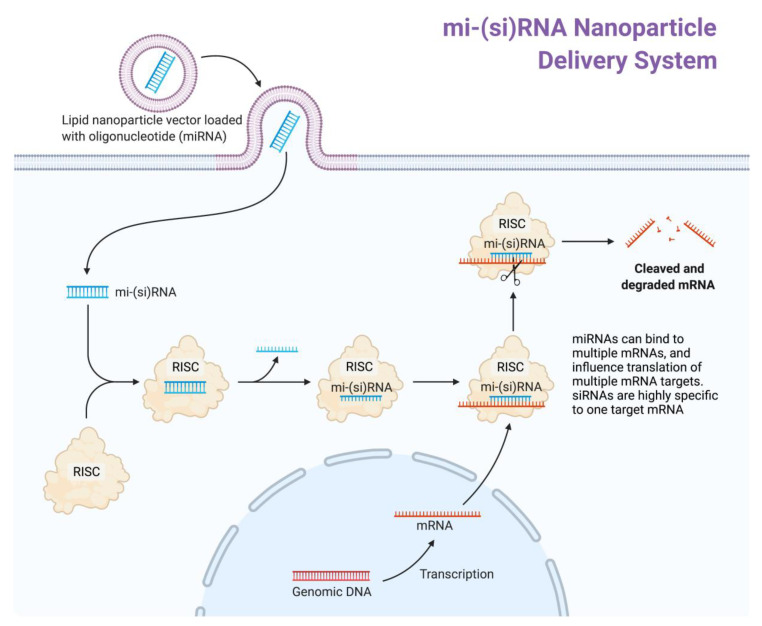
Concept of RNA-based drug delivery to target tissues. Mi-(si)-RNA is delivered by lipid particles, which can be incorporated by target cells via endocytosis. The delivered oligonucleotide can be taken up by the RNA-induced silencing complex (RISC), a ribonucleoprotein-complex. The introduced RNA serves as a template for RISC to bind a  complementary intracellular mRNA transcript. Once a complementary mRNA binds to RISC, the complex induces cleaving and degradation of the target mRNA strand, effectively “silencing” mRNA expression of the target.

The delivery of miRNA molecules to the target organ is as critical to the success of these novel drugs as the identification of miRNA targets. With short strand RNAs being transported physiologically in exosomes and similar small vesicles, current miRNA-and siRNA-derived drugs apply this principle by delivering the oligonucleotides encapsulated in lipid spheres, with high efficiency (Figure 1) [63].

Reprinted from “siRNA Nanoparticle Delivery System”, by BioRender.com (2020). Retrieved from https://app.biorender.com/biorender-templates (accessed on 15 October 2021).

## 4. Conclusions

Since their discovery in the 1990s, our knowledge on miRNAs has rapidly increased, leading to the understanding that virtually every cellular process is modulated by miRNAs. In the cardiovascular field, a large body of evidence is available, i.e., different types of heart disease go along with major changes in miRNA expression profiles. Specific miRNAs have been identified in conditions, such as acute myocardial ischemia, and several types of non-ischemic cardiomyopathies that are promising candidates for biomarkers. However, the validity of applying miRNAs for this purpose is limited by often-heterogeneous results, with little overlap between reported miRNA profiles in between studies and, therefore, possibly, low reproducibility. This problem may be addressed in the future, with larger, unbiased, and methodologically aligned studies for miRNA profiling. Therefore, to date, there are no miRNA-based biomarkers that can outperform established, mostly protein-based assays. Regarding miRNA-based drugs—while the development of effective therapeutics has been hampered by the complex mechanisms and interactions between miRNAs and gene expression, as well as the development of effective delivery systems of oligonucleotide-based drugs, the development of efficient lipid-particle based delivery vehicles, and tremendous research efforts into mechanistic details of miRNA-regulated pathways, have recently led to the first oligonucleotide-based drugs entering clinical trials, or, in regards to siRNA-based molecules, have even gained approval for clinical use. While it is yet to be determined which technology would be the most applicable approach, RNA-based therapeutics show great promise at becoming an invaluable new drug class.

## Figures and Tables

**Table 1:** Potential miRNA-based biomarkers for heart disease.

CMP	Associated miRNA	Evidence	Ref.
Ischemic Heart Disease	miR-133; miR-499; miR-1; miR-208 (all blood)	Comparable performance in ACS to troponin; potential benefit of multi-miRNA panel; advantageous kinetics (remain stable in circulation >24 h)	[8]
HCM	miR-29a/c (blood); (miR-373; miR-371-3p, miR-34b; miR-452)-Cluster (myocardium)	Preliminary, small studies; identify fibrosis, identify HOCM phenotype	[9,10]
Amyloidosis	miR-339-3p; miR-150-5p (blood)	Exploratory, small studies; identify ATTRwt; identify symptomatic ATTRm	[11,12]
Sarcoidosis	miR-7-1-3p, miR-32-3p, miR-211-5p; miR-126 and miR-223 (all blood)	Small study, identified miRNAs associated with multiple cardiovascular mechanisms (marker of myocardial cell death) or immunological function	[13,14]
ARVC	miR-21-5p and miR-135b (myocardium); miR-144-3p, miR-145-5p, miR-185-5p, miR-494 (blood); miR-122-5p, miR-133a-3p, miR-133b, miR-142-3p, miR-182-5p, and miR-183-5p (blood and myocardium)	Linked miRNA regulatory function to Wnt pathway, mechanistic insights. miRNA-signatures with reportedly excellent discriminatory powers to distinguish between ARVC and controls	[15,16,17,18]
Myocarditis	Various miRNA- clusters for immune regulation, apoptosis, cardiomyocyte specific miRNAs (necrosis), fibrosis; leukocyte derived miRNAs (miR-Chr8:96) (blood)	miRNA clusters established that regulate immune response, leucocyte activation. MyomiRs released in circulation as markers of cardiomyocyte cell death	[19]
Stress CMP	miR-16, miR-26a (blood)	Mechanistic data on miR-16 and miR-26a mediated catecholaminergic stress CMP; promising biomarkers, potential therapeutic approach (miR-16/26a inhibitors for cardiogenic shock caused by stress CMP?)	[20,21]

## Data Availability

Data sharing not applicable. No new data were created or analyzed in this study.
